# Declined Organs for Liver Transplantation: A Right Decision or a Missed Opportunity for Patients with Hepatocellular Carcinoma?

**DOI:** 10.3390/cancers15051365

**Published:** 2023-02-21

**Authors:** Vladimir J. Lozanovski, Said Adigozalov, Elias Khajeh, Omid Ghamarnejad, Ehsan Aminizadeh, Christina Schleicher, Thilo Hackert, Beat Peter Müller-Stich, Uta Merle, Susanne Picardi, Frederike Lund, De-Hua Chang, Markus Mieth, Hamidreza Fonouni, Mohammad Golriz, Arianeb Mehrabi

**Affiliations:** 1 Department of General, Visceral and Transplant Surgery, University Hospital Heidelberg, 69120 Heidelberg, Germany; 2 Liver Cancer Center Heidelberg, University Hospital Heidelberg, 69120 Heidelberg, Germany; 3 German Organ Procurement Organization (Deutsche Stiftung Organtransplantation, DSO), 60594 Frankfurt, Germany; 4 Department of General, Visceral and Thoracic Surgery, University Hospital Hamburg Eppendorf, 20251 Hamburg, Germany; 5 Department of Internal Medicine IV, University Hospital Heidelberg, 69120 Heidelberg, Germany; 6 Department of Anesthesiology, University Hospital Heidelberg, 69120 Heidelberg, Germany; 7 Department of Radiology, University Hospital Heidelberg, 69120 Heidelberg, Germany

**Keywords:** Eurotransplant, extended donor criteria, liver transplantation, declined organs, MELD, extended right lobe liver transplantation, hepatocellular carcinoma, HCC

## Abstract

**Simple Summary:**

Every declined organ is a missed opportunity that increases mortality on the waiting lists. There are many reasons why an organ may be declined for transplantation. To better understand this complex situation, we analyzed the factors involved in organ allocation in our transplant center. This study has shown that 50% of potentially suitable organs are declined. This indicates that decision-making is not standardized during allocation, and that whether to accept or decline an organ is at the discretion of the transplant teams, who evaluate the organ risk based on the information available. Our results show that, while an organ might be unsuitable for one recipient, it might be suitable for another. We also show that patient care can be improved and emphasize the need for optimized allocation protocols to avoid unnecessary declination of organs. This is particularly relevant to major extended donor criteria grafts, which are becoming the new “standard” and need specific allocation policies.

**Abstract:**

Background: Liver transplantation is the only promising treatment for end-stage liver disease and patients with hepatocellular carcinoma. However, too many organs are rejected for transplantation. Methods: We analyzed the factors involved in organ allocation in our transplant center and reviewed all livers that were declined for transplantation. Reasons for declining organs for transplantation were categorized as major extended donor criteria (maEDC), size mismatch and vascular problems, medical reasons and risk of disease transmission, and other reasons. The fate of the declined organs was analyzed. Results: 1086 declined organs were offered 1200 times. A total of 31% of the livers were declined because of maEDC, 35.5% because of size mismatch and vascular problems, 15.8% because of medical reasons and risk of disease transmission, and 20.7% because of other reasons. A total of 40% of the declined organs were allocated and transplanted. A total of 50% of the organs were completely discarded, and significantly more of these grafts had maEDC than grafts that were eventually allocated (37.5% vs. 17.7%, *p* < 0.001). Conclusion: Most organs were declined because of poor organ quality. Donor-recipient matching at time of allocation and organ preservation must be improved by allocating maEDC grafts using individualized algorithms that avoid high-risk donor-recipient combinations and unnecessary organ declination.

## 1. Introduction

Liver transplantation is the only promising treatment for end-stage liver disease and patients with hepatocellular carcinoma (HCC). Although efforts have been made to expand the pool of available organs without compromising patient safety and ethical standards, too many organs are still declined for transplantation. Between 2016 and 2020, 10,113 liver donors were reported to Eurotransplant (ET), but 2428 (24%) of these organs were not used. During this time, 2116 (~30%) of all listed transplant candidates in ET died while waiting for an organ, and 462 candidates dropped out of the waiting list because they became too sick to receive a transplant [1,2]. Similar trends have also been reported in the United States, where transplant candidates received a median of five liver offers before they died or dropped out of the waiting list, and 84% of the candidates who were removed from the waiting list because they became too sick to receive a transplant or died while waiting for an organ, received at least one offer before the terminal event [3,4]. These patients received a median of two organ offers more than patients who were eventually transplanted, and the organs were refused, most commonly because of the donor quality or age [4]. Similar trends have been reported in Germany [5].

Efforts to improve access to transplantation have revealed differences in organ donation and organ acceptance practices [6]. In Germany, donor and donor organ assessment is standardized by the German medical chamber (Bundesärztekammer (BÄK)) and by the German Organ Procurement Organization (Deutsche Stiftung Organtransplantation (DSO)). For liver transplantation, ET allocation algorithms are also included in the BÄK guidelines. Despite these detailed guidelines on organ allocation and the fact that all relevant information on organs procured in Germany is usually fully available to the transplant centers, the decisions are often subjective during allocation, and whether to accept or decline an organ offer is at the discretion of the local transplant team [7]. Transplant surgeons typically prefer to avoid risk and decide whether to allocate a donor organ based on short-term results [8]. Instead of accepting an organ that may be associated with a high risk of poor postoperative outcome, transplant surgeons tend to wait longer for a better graft, disregarding the potential benefits the organ may offer, and that there is no guarantee that a better organ will become available before the transplant candidate dies or is removed from the waiting list because the disease (e.g., HCC) progressed beyond the transplantation criteria [9,10,11].

There are many reasons why an organ may be declined for transplantation. To better understand this complex situation, we analyzed the factors involved in organ allocation in our transplant center. We evaluated how organs were allocated for transplantation, why these organs were declined, and what happened to them after they were declined.

## 2. Materials and Methods

All livers from brain-dead donors that were declined for transplantation by our center between 2016 and 2020 were reviewed. Data on donor and recipient characteristics, allocation procedures, reasons for declining, and the fate of declined organs were collected from a prospective database, a transplant registry, written and electronic medical records, ET allocation protocols, and DSO protocols. Organ offers were analyzed separately from organs that may have been offered more than once (one offer one case). The reasons why an organ was declined were extracted from handwritten and electronic allocation protocols and no organ offer was excluded from the analysis. The Ethics Committee of the University of Heidelberg approved the analysis (S-548/2012).

### 2.1. Organ Allocation

Offered organs were characterized as primary, extended, or rescue allocations [12].

#### 2.1.1. Primary Allocation

Donor organs are reported to ET, which prioritizes organ allocation based on urgency and model for end-stage liver disease (MELD) scores [12]. Liver transplant candidates are ranked on a match list and ET offers the organ to the first candidate on this list and contacts the corresponding center (primary allocation), where the local transplant surgeon decides whether to accept the organ or not. Two candidates receive the same organ offer, but the higher-ranked candidate is prioritized. This decision has to be made within 30 min and the reason for declining an organ is recorded [12].

#### 2.1.2. Extended Allocation

If the organ cannot be delivered to the transplant center in time or if the organ is refused by three different centers, ET offers the organ to regional transplant centers (patient-oriented extended allocation). The regional centers have to choose two patients from their waiting list and report them to ET within 30 min. The offer goes to the highest-ranked candidate [12].

#### 2.1.3. Rescue Allocation

If the extended allocation fails, the organ is offered to further centers on a first-come first-served basis (center-oriented rescue allocation). If no suitable recipient can be found within ET, the organ is offered to other countries. Together with the local coordinator, ET can decide to withdraw the organ [12].

#### 2.1.4. Assessing Transplant Candidate Condition

Transplant candidate condition was assessed using the laboratory model for end-stage liver disease (labMELD) score. A cut-off value of 20 differentiated between low-risk (labMELD < 20) and high-risk (labMELD ≥ 20) transplant candidates, as previously reported [13]. Transplant candidates with standard exceptional MELD (eMELD) scores received MELD points that started at a fixed initial value. The eMELD score was upgraded as long as the defining condition persisted [14]. The matchMELD score was calculated as the highest labMELD or eMELD score.

### 2.2. Reasons for Declining Organs for Transplantation

A categorization system was developed to characterize the reasons for declining an organ. Multiple reasons for declining were possible and different offers may have been declined for the same reason.

#### 2.2.1. Major Extended Donor Criteria

Donor age > 65 years, biopsy proven macrovesicular steatosis >40%, estimated prolonged cold ischemia time (CIT) > 14 h, and extended right lobe (ERL) livers were considered major extended donor criteria (maEDC) [15,16,17,18,19]. Organs declined because of maEDC were analyzed separately.

#### 2.2.2. Size Mismatch and Vascular Problems

Organs were declined because of size mismatch if the body mass index (BMI) of the donor and recipient were significantly different. Body surface area (BSA) was calculated using the formula BSA = weight (kg) ^0.425^ × height (cm) ^0.725^ × 0.007184. The body surface area index (BSAi) was calculated using the formula BSAi = donor BSA/recipient BSA, as previously reported [20]. BMI and BSAi values were compared between organs declined because of size mismatch and organs declined for other reasons.

Vascular problems considered valid reasons for declining an organ were short graft vessels, mismatch between vascular diameter of the donor and recipient, and vascular injury during the organ procurement phase.

#### 2.2.3. Medical Reasons and Risk of Disease Transmission

Organ offers from donors with severely abnormal liver function tests (transaminases more than three times the normal level [46–50 U/L] and bilirubin >3 mg/dL), severe hypernatremia (>165 mmol/L), livers from hemodynamically instable donors or from donors who have been reanimated over longer periods, and offers from donors with multiple comorbidities or from donors who drowned in salty water were considered high-risk and were declined. Organ offers from donors with recent malignancy and/or infectious diseases were declined because they were categorized as high or unacceptable risk for transplantation according to the Guide to the Quality and Safety of Organs for Transplantation or because the risk-benefit analysis did not justify acceptance for the recipient [21].

#### 2.2.4. Other Reasons

Rescue allocation organs were declined if the recipient’s blood group was incompatible with that of the donor or if the recipient was not transplantable at the time of the allocation.

### 2.3. Fate of Declined Organs

The fate of the declined organs was also analyzed and the reasons for declining an organ were compared between the group of further allocated and transplanted organs and the group of completely discarded organs.

### 2.4. Statistical Analysis

IBM SPSS Statistics for Windows, Version 22.0 (IBM Corp., released 2013, Armonk, NY, USA) was used for statistical analyses. Continuous data are expressed as mean ± standard deviation (SD) or median and range, and categorical variables are shown as percentages. Independent *t*-test and Mann-Whitney U test for non-normally distributed data were used to compare continuous variables between groups. Categorical variables were analyzed using the Pearson chi-square test or Fisher exact test. A two-sided *p* < 0.05 was considered statistically significant.

## 3. Results

### 3.1. Allocation Type, Donor, and Recipient Characteristics

Our transplant center was offered 1311 livers, and we declined 1086 organs that were offered 1200 times (462 primary, 180 extended, and 524 rescue allocation offers). Allocation type was missing for 34 organ offers. Of the 1086 offered organs, 863 were declined once, 99 twice, seven three times, and one four times. The median donor age was 59 years (range 0–96 years), and 56% of donors were male. The mean donor BMI was 27.21 ± 6.33 kg/m^2^, and the mean recipient BMI was 25.9 ± 5.82 kg/m^2^. The median transplant candidate age was 52 years, and 51% were male (Table 1).

### 3.2. Waiting List Dynamics

During the study period, 203 liver transplantations were performed, and 255 of 600 transplant candidates on the waiting list either recovered or asked to be removed from the waiting list. Ninety-one transplant candidates died while waiting for transplantation, and 36 patients were removed from the waiting list because their disease had progressed beyond the transplantation criteria. Fifteen patients were referred to another center adjacent to their new place of residence.

The mean labMELD score was 20.2 ± 9.79 and the mean eMELD score was 27.41 ± 6.15 (Table 1). In non-HCC patients listed for transplantation, the mean labMELD score was 21 ± 9.7, and 111 of 539 non-HCC patients were removed from the waiting list or died of their disease. Each non-HCC patient received at least one organ offer (median, 2; range, 1–26). A median of 9 days (range 1–437) passed between the last organ offer and death or removal from the waiting list in these candidates. In transplant candidates with HCC, the mean eMELD score was 27.41 ± 6.2, and 22 of 61 HCC patients were removed from the waiting list because they did not fulfill the criteria for transplantation anymore or died of their disease. Each transplant candidate with HCC received at least one organ offer (median, 2; range, 1–24). A median of 35 days (range 2–113) passed between the last organ offer and death or removal from the waiting list.

### 3.3. Reasons for Declining an Organ for Transplantation

#### 3.3.1. Major Extended Donor Criteria

A total of 336 (30.9%) of 1086 livers (368 of 1200 offers) with maEDC were declined. We declined 45 (4.1%) livers from older donors, 192 (17.7%) macroscopic fatty livers or livers with biopsy proven macrovesicular steatosis, 72 (6.6%) livers with a long estimated conservation time, and 48 (4.4%) split livers (Table 2). Interestingly, none of the organs from donors older than 65 years were split livers.

The labMELD and eMELD scores were similar between transplant candidates who were offered livers from donors older than 65 years and transplant candidates who were offered livers from younger donors.

Donors of steatotic livers were significantly older than donors of non-steatotic livers. Fewer steatotic livers with a longer estimated CIT were offered than non-steatotic livers with a longer CIT. None of the offered steatotic livers were split livers and most of these organs were primarily offered to transplant candidates with lower labMELD scores (Table 2).

Donors of split livers were significantly younger than donors of full-size livers, and the estimated CIT was significantly longer in split livers. The labMELD scores between the groups were comparable, but split livers were predominantly allocated to female patients and patients with higher eMELD scores (Table 2).

#### 3.3.2. Size Mismatch and Vascular Problems

A total of 386 (35.5%) size mismatch livers of 1086 livers (427 of 1200 organ offers) were declined, and 6 livers (seven offers) with vascular problems were declined. The BMI and BSAi of both donor and transplant candidate were known in 669 organ allocation offers. As expected, BMI differences were greater between donors and candidates in the size mismatch group than in the control group (6.32 ± 5.96 vs. 4.10 ± 3.73, *p* < 0.001) (Table 3). BSAi extremes (<0.78 and >1.24) were significantly higher in the size mismatch group than in the control group (9.6% vs. 4.9%, *p* < 0.001, and 26.3% vs. 2.6%, *p* < 0.001, respectively) indicating an optimal estimation of the donor-recipient size mismatch. The BSAi of offered livers was in the normal range (0.78–1.24) for 539 organs. However, 180 organ offers were declined because of size mismatch despite the normal range of BSAi (0.78–1.24), indicating a suboptimal estimation of the donor-recipient size mismatch in 64.1% of the 281 organ offers that were declined based on BMI size mismatch estimation (Table 3).

#### 3.3.3. Medical Reasons, Risk of Disease Transmission, and Other Reasons

A total of 172 (15.8%) of 1086 livers (188 of 1200 organ offers) were declined because of medical reasons and risk of disease transmission, and 225 (20.7%) organs (253 offers) were declined because of other reasons.

### 3.4. Fate of Declined Organs

#### 3.4.1. Further Allocated and Transplanted Organs

**Figure 1:**
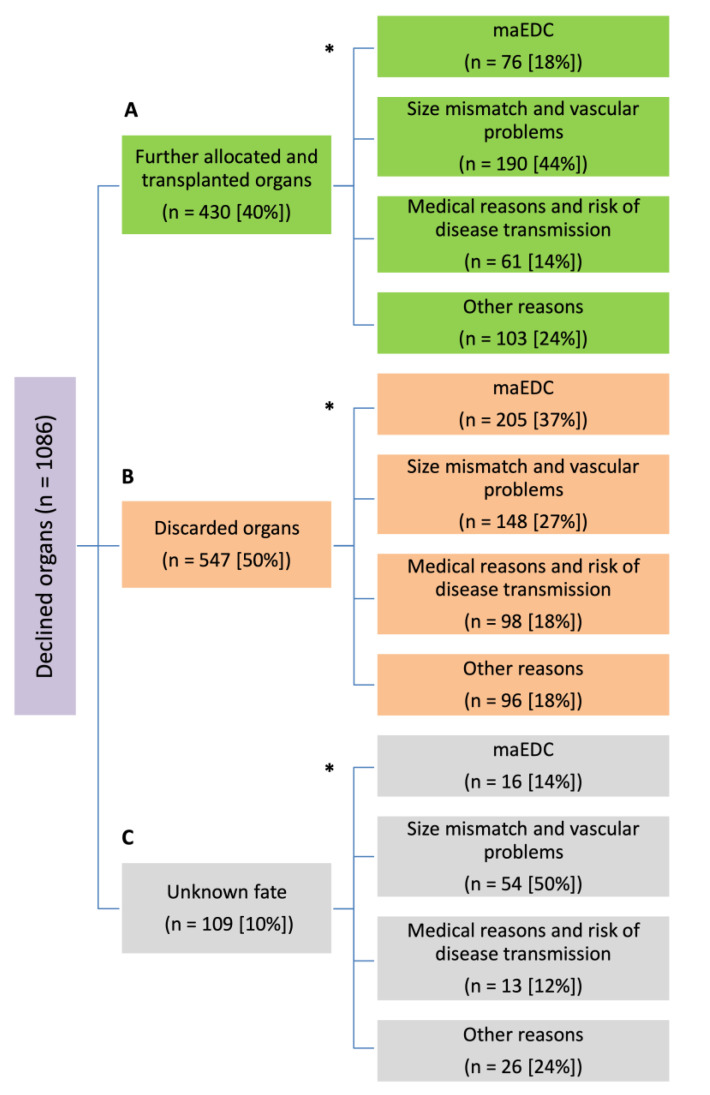
Flow diagram of declined organs (n = 1086). Fate of the declined organs (A–C) and reasons for declining an organ (*). maEDC, major extended donor criteria.

**Figure 2:**
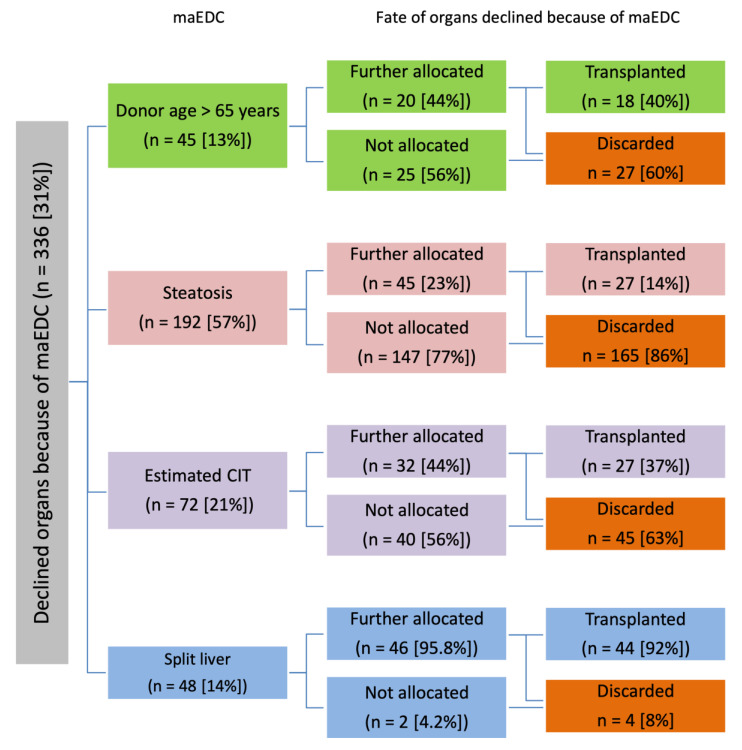
Flow diagram and fate of organs declined because of major extended donor criteria (maEDC; n = 336; 31% of all declined organs). Multiple reasons for declining were possible, and different offers may have been declined for the same reason. CIT, cold ischemia time; maEDC, major extended donor criteria.

A total of 430 (40%) of 1086 declined organs were allocated and transplanted in our center (n = 32; 7%) or in another transplant center (n = 398; 93%) (Figure 1A). Of these 430 organs, 76 (18%) were declined because of maEDC, 190 (44%) because of size mismatch and vascular problems, 61 (14%) because of medical reasons and risk of disease transmission, and 103 (24%) because of other reasons. The fate of organs that were declined because of maEDC is summarized in Figure 2.

#### 3.4.2. Discarded Organs

A total of 547 (50%) of 1086 declined livers were completely discarded; 498 (91%) of these were not further allocated, and 49 (9%) livers were transferred and then discarded in another center. Of these 547 organs, 205 (37%) were declined because of maEDC, 148 (27%) because of size mismatch and vascular problems, 98 (18%) because of medical reasons and risk of disease transmission, and 96 (18%) because of other reasons (Figure 1B). The fate of organs that were discarded because of maEDC is summarized in Figure 2.

#### 3.4.3. Unknown Fate

The fate of 109 (10%) of 1086 declined livers were unknown. Of these 109 organs offered from abroad, 16 (14%) were declined because of maEDC, 54 (50%) because of size mismatch and vascular problems, 13 (12%) because of medical reasons and risk of disease transmission, and 26 (24%) because of other reasons (Figure 1C). The fate of organs that were declined because of maEDC is summarized in Figure 2.

The reasons for organ declination and the fate of declined organs are summarized in Table 4. Significantly more grafts that were completely discarded had maEDC than grafts that were eventually allocated (37.5% vs. 17.7%, *p* < 0.001). Size mismatch and vascular problems were significantly more prevalent in the group of allocated organs than in the group of discarded organs (44.2% vs. 27.1%, *p* < 0.001 and 24% vs. 17.6%, *p* = 0.016, respectively). The fate of organs that were declined because of maEDC is summarized in Figure 2.

## 4. Discussion

Since 1967, patient-oriented ET has facilitated international organ exchange for transplantation within Europe [12]. A shortage of organs has driven transplant centers to accept maEDC livers for transplantation in recent years [17]. These organs are acceptable for adult recipients with lower labMELD scores and recipients with HCC who are generally in a better condition because they do not affect patient survival [15,16,17,18,19]. However, maEDC livers are associated with postoperative complications and affect graft survival, so careful donor-recipient matching is important [17,22]. Still, far too many organs are declined [4,5].

Every organ that is declined is a missed opportunity that increases mortality on the waiting lists. This study has shown that 50% of potentially suitable organs are declined. This indicates that decision-making is not standardized during allocation, and that whether to accept or decline an organ is at the discretion of the local transplant teams, who evaluate the organ risk based on the information available. Our results show that while an organ might be unsuitable for one recipient, it might be suitable for another. We also show that patient care can be improved and emphasize the need for optimized allocation protocols to avoid unnecessary declination of organs. This is particularly relevant to HCC patients and to maEDC grafts, which are becoming the new “standard” and need specific allocation policies.

The median donor age has increased in ET, and >33% of the donors are now ≥65 years old [23,24]. The risk of graft loss increases linearly with donor age, but survival benefits have been reported in recipients of grafts from older donors, especially older recipients and recipients with HCC, who seem to be less affected by advanced donor age [16,23,24,25]. The present study showed that the acceptance rate of livers from older donors is high, which may reflect the pressures of organ shortage.

Macrovesicular steatosis >40% is the strongest predictor of graft loss, especially in combination with advanced donor age or longer CIT [17]. However, HCC patients with labMELD scores <20 seem to be less affected by steatotic livers [16]. In our study, steatotic livers were primarily offered to low-risk transplant candidates; however, most steatotic livers were completely rejected, even after being allocated to other centers. A prolonged CIT reduces graft survival, but this depends strongly on the underlying disease. A longer CIT reduces survival more in patients with hepatitis C-related cirrhosis; in contrast, patients with HCC and alcoholic cirrhosis with labMELD scores <20 can tolerate a longer CIT without significant impact on survival. This suggests that grafts with longer CITs should preferentially be allocated to these recipients [15,19]. The present study showed that over two thirds of organs declined because of CIT were completely rejected.

Most ERL grafts were declined because of donor-recipient size mismatch. However, ERL livers were also declined because they were offered to high-risk recipients. On the one hand, this decision is justified as ERL grafts have been associated with higher vasculobiliary complication rates, re-transplantation rates, and lower graft survival [18]. On the other hand, patient survival has been shown to be unaffected by ERL livers, indicating that these grafts can be used safely [18,19]. However, only optimal, high-quality organs are considered for split-liver transplantations, and a short CIT is essential for therapy success [19,26,27]. The allocation process is very complex and the estimation of CIT before organ procurement is imprecise. Still, the conservation time is ~50% longer in split liver transplantation than in full size transplantation. With this in mind, ex situ split livers should be allocated to patients at the center performing the split procedure, whereas in situ split grafts can be allocated to different centers. In this way, conservation time can be reduced and allocation and prioritizing bias can be avoided [18,19]. Split liver was considered a maEDC, but it was not a primary reason for declining an organ in our study. This may explain the high transplantation rate of 92%, but it may also reflect the pressures of organ shortage.

We recently suggested an allocation algorithm that balances the number of maEDC with the recipient’s condition [17]. According to this algorithm, grafts with more than one maEDC should be allocated to low-risk transplant candidates and/or transplant candidates with HCC or alcoholic liver cirrhosis who are less vulnerable to the effects of maEDC [15,16,17,19]. This leaves non-maEDC grafts free for recipients who need them, such as patients with hepatitis C-related cirrhosis or high-risk patients with labMELD scores of > 20. Most of the offered maEDC grafts were completely discarded, even after being allocated to other centers. This emphasizes the poor organ quality in ET and shows that maEDC organs are still considered unsuitable for transplantation and are discarded most of the time [28]. However, this might also reflect a suboptimal allocation of maEDC grafts to suitable recipients, possibly because accurate maEDC data were missing at the time of allocation. Donor age and steatosis cannot be modified, but livers from older donors or fatty livers can be allocated more adequately. On the one hand, inspection-based liver assessment is a poor predictor of higher-grade steatosis [29,30]. On the other hand, waiting for pathology results after organ procurement prolongs CIT unnecessarily. Each additional hour of cold storage increases the risk of 1-year graft loss by 3.4% after full-size liver transplantation and by 10% after extended right lobe liver transplantation (ERLT) [15,19,31]. Therefore, to accurately assess the grade of steatosis, a biopsy should be taken and the steatosis grade of the donor liver (alone or in combination with other maEDC, especially CIT) should be considered even before allocation to avoid high-risk donor-recipient combinations and to ensure optimal recipients are selected for each organ. This would reduce the number of organs that are declined. Ideally, an organ should be transplanted into the intended recipient, but there should always be another candidate close to the transplant center with an uncomplicated anatomy to whom the liver can be reallocated without prolonging cold storage [32]. Other centers were willing to accept some maEDC grafts that we had declined, such as organs from older donors and split livers, possibly because their candidates were more suitable for these grafts, or possibly because these centers used machine perfusion techniques that reduce ischemia reperfusion injury and increase organ viability [33,34,35,36,37,38,39]. Although we cannot confirm that machine perfusion was used in these reallocations, our results suggest that optimizing allocation logistics and organ preservation can solve the problems of prolonged CIT, which is the only modifiable maEDC. Deeper understanding of HCC biology and artificial intelligence could ensure optimal organ allocation [40,41].

Before declining organs because of size mismatch, more suitable parameters should be considered. Fukazawa et al. developed a model that estimated 3-year graft survival based on the BSAi in a collective of 24,509 patients [20]. The authors suggested a BSAi range of 0.78–1.24 for avoiding adverse outcomes associated with size mismatch [20]. In agreement with this, we also found that the BSAi may be a more sensitive indicator of size mismatch than BMI, as well as the fact that we may have been too cautious and declined too many organs because of size mismatch in the past. Most of the organs we have declined because of size mismatch were transplanted elsewhere, and we have now integrated the BSAi into our recipient assessment and organ allocation protocols.

Only one third of the grafts that were declined for medical reasons were transplanted elsewhere, confirming that our decision to decline them was solid. However, it is not clear whether the eventual recipients of these organs were in fact suitable. This is a limitation of our study. Most of the competitive organ offers were accepted elsewhere because there was no matching recipient on our waiting list, or our recipients were non-transplantable. This identifies important communication gaps between transplant institutions that need to be overcome in the future to ensure optimal organ allocation.

LabMELD scores among the listed non-HCC transplant candidates at the time of organ offer and the time that passed between their last organ offer and their death or removal from the waiting list suggest that these candidates were more ill than their labMELD score indicated at the time of organ offer. This shows that high-risk graft offers were declined and that transplant surgeons either waited for the clinical condition to improve or hoped for a better organ offer. This confirms the subjective nature of the decision-making process.

## 5. Conclusions

The numbers of organs that were further allocated and transplanted shows that the ET allocation model provides equal opportunities for all transplant candidates. However, most organs were declined because of organ quality and for reasons that cannot be modified. High-quality donor organs are scarce, so transplant surgeons must make high-risk subjective decisions on whether to accept a potentially risky organ or wait for a better one that may not come before the patient dies or has to be removed from the waiting list because the disease, especially HCC, has progressed beyond the transplant criteria. To help with this challenge, donor-recipient matching at time of allocation and organ preservation must be improved. This would allow the avoiding of unnecessary organ transport and reduce costs and could be achieved by allocating maEDC grafts using individualized algorithms that avoid high-risk donor-recipient combinations and unnecessary organ declination. Importantly, the impact of CIT on post-transplant outcomes could be improved by managing CIT during organ procurement and by promoting communication between institutions during organ allocation. More transparency regarding decisions to accept or decline an organ may also help standardize organ allocation, and the donor pool could be expanded by accepting the policy that everybody is a donor unless they opt out.

## Figures and Tables

**Table 1:** Demographic characteristics and clinical donor and recipient parameters.

Donor Characteristics				
	Mean	Median	SD	Range (min–max)
Age (years)	57	59	20	0–96
BMI (kg/m^2^)	27.21	26.12	6.33	11–68
Weight (kg)	81	80	22.71	3–190
Height (cm)	171	174	17	52–200
Gender Female (n, %) Male (n, %)	475 (44) 611 (56)			
**Recipient characteristics**				
	Mean	Median	SD	Range (min–max)
Age (years)	49	52	12	15–74
BMI (kg/m^2^)	25.94	25.03	5.82	17–53
Weight (kg)	77.5	75	22.34	42–185
Height (cm)	172	170	10	147–205
labMELD	20.2	18	9.79	6–40
eMELD	27.41	27.53	6.15	2–40
MatchMELD	23.86	23	9.11	7–40
Gender Female (n, %) Male (n, %)	330 (49) 346 (51)			

BMI, body mass index; labMELD, laboratory model for end-stage liver disease score; eMELD, standard exception model for end-stage liver disease score; matchMELD, either eMELD or labMELD score (the higher value was chosen for allocation purposes).

**Table 2:** Comparison of donor and recipient characteristics in cases of organs declined because of major extended donor criteria (maEDC) and organs declined for other reasons.

Donor Age > 65 Years			
	Yes (n = 45)	No (n = 1041)	*p*
Female donor (n, %)	31 (68.9)	443 (42.6)	<0.001
Donor age (mean ± SD)	81 ± 7.2	56 ± 19.2	<0.001
Donor BMI (mean ± SD)	26.2 ± 6	27.3 ± 6.3	0.25
Liver steatosis (n, %)	2 (4.4)	190 (18.3)	0.015
Split organs (n, %)	0 (0)	48 (4.6)	0.258
Estimated CIT > 14 h (n, %)	4 (8.9)	68 (6.5)	0.534
Female recipient (n, %)	21 (46.7)	273 (26.2)	0.005
Recipient age (mean ± SD)	46 ± 14	49 ± 12.5	0.174
Recipient BMI (mean ± SD)	26.4 ± 5.6	25.7 ± 5.6	0.53
labMELD (mean ± SD)	19.8 ± 8.7	20.8 ± 10	0.582
eMELD (mean ± SD)	28.2 ± 6	27.5 ± 5.9	0.774
**Liver steatosis**			
	**Yes (n = 192)**	**No (n = 894)**	***p***
Female donor (n, %)	85 (44.3)	389 (43.5)	0.873
Donor age (mean ± SD)	62 ± 15.8	55 ± 20.1	<0.001
Donor age > 65 years (n, %)	81 (42.2)	294 (32.9)	0.015
Donor BMI (mean ± SD)	29.5 ± 6.7	26.7 ± 6.1	<0.001
Split organs (n, %)	0 (0)	48 (5.4)	<0.001
Estimated CIT > 14 h (n, %)	6 (3.1)	66 (7.4)	0.036
Female recipient (n, %)	25 (29.1)	269 (52.7)	<0.001
Recipient age (mean ± SD)	52 ± 9	49 ± 13	0.012
Recipient BMI (mean ± SD)	27.7 ± 5.1	25.4 ± 5.6	<0.001
labMELD (mean ± SD)	18.3 ± 8.3	21.2 ± 10.1	0.007
eMELD (mean ± SD)	25.0 ± 3.2	27.8 ± 6.1	0.106
**Long estimated CIT**			
	**Yes (n = 72)**	**No (n = 1014)**	***p***
Female donor (n, %)	27 (37.5)	447 (44.1)	0.325
Donor age (mean ± SD)	55 ± 17.3	57 ± 19.7	0.361
Donor age > 65 years (n, %)	22 (30.6)	353 (34.8)	0.522
Donor BMI (mean ± SD)	26.4 ± 4.4	27.3 ± 6.4	0.141
Liver steatosis (n, %)	6 (8.3)	186 (18.3)	0.036
Split organs (n, %)	8 (11.1)	40 (3.9)	0.011
Female recipient (%)	12 (40)	282 (49.8)	0.35
Recipient age (mean ± SD)	47 ± 13.7	49 ± 12.5	0.309
Recipient BMI (mean ± SD)	25.6 ± 4.4	25.8 ± 5.6	0.902
labMELD (mean ± SD)	21.7 ± 12	20.7 ± 9.8	0.676
eMELD (mean ± SD)	32.4 ± 7.3	27.3 ± 5.8	0.090
**Split livers**			
Female donor (n, %)	**Yes (n = 48)**	**No (n = 1038)**	***p***
Female donor (n, %)	20 (41.7)	454 (43.7)	0.882
Donor age (mean ± SD)	36 ± 15.4	58 ± 19.2	<0.001
Donor age > 65 years (n, %)	0 (0)	375 (36.1)	<0.001
Donor BMI (mean ± SD)	23.1 ± 3.2	27.4 ± 6.4	<0.001
Liver steatosis (n, %)	0 (0)	192 (18.5)	<0.001
Estimated CIT > 14 h (n, %)	8 (16.7)	64 (6.2)	0.011
Female recipient (n, %)	25 (65.8)	269 (48.2)	0.044
Recipient age (mean ± SD)	44 ± 14	49 ± 12.4	0.010
Recipient BMI (mean ± SD)	24.8 ± 7.5	25.8 ± 5.4	0.289
labMELD (mean ± SD)	21 ± 11.7	20.7 ± 9.8	0.864
eMELD (mean ± SD)	31.3 ± 5.5	26.9 ± 5.8	0.006

BMI, body mass index; CIT, cold ischemia time; labMELD, laboratory model for end-stage liver disease score; eMELD, standard exception model for end-stage liver disease score; SD, standard deviation.

**Table 3:** BSAi analysis and comparison between organ offers declined because of size (BMI) mismatch and offers declined because of reasons other than size mismatch.

	Size Mismatch n = 281	Reasons to Decline Other than Size Mismatch n = 388	*p*
BSAi			
BSAi < 0.78 (n, %)	27 (9.6)	19 (4.9)	<0.001
BSAi = 0.78–1.24 (n, %)	180 (64.1)	359 (92.5)	
BSAi > 1.24 (n, %)	74 (26.3)	10 (2.6)	
BMI			
BMI (mean ± SD)	6.32 ± 5.96	4.1 ± 3.73	<0.001
BMI (median (range))	5.12 (0.11–35.88)	3.27 (0.02–31.42)	

BSAi, body surface area index; BMI, body mass index.

**Table 4:** Reasons for declining an organ and comparison between organs that were reallocated and organs that were discarded.

Reasons for Declining an Organ	Further Allocated and Transplanted Organs n = 430	Discarded Organs n = 547	*p*
maEDC (n, %)	76 (17.7%)	205 (37.5%)	<0.001
Size mismatch and vascular problems (n, %)	190 (44.2%)	148 (27.1%)	<0.001
Liver function and medical issues (n, %)	61 (14.2%)	98 (17.9%)	0.138
Other reasons (n, %)	103 (24%)	96 (17.6%)	0.016

maEDC, major extended donor criteria.

## Data Availability

The data presented in this study are available on request from the corresponding author.

## Abbreviations

BÄK, Bundesärztekammer; BMI: body mass index; BSA, body surface area; BSAi, body surface area index; CIT, cold ischemia time; DSO, German Organ Procurement Organization (Deutsche Stiftung Organtransplantation); EDC, extended donor criteria; eMELD, exceptional model of end-stage liver disease; ERLT, extended right lobe liver transplantation; ET, Eurotransplant; HCC, hepatocellular carcinoma; maEDC, major extended donor criteria; MELD, model of end-stage liver disease; SD, standard deviation.
